# Leaving no one behind? Addressing inequitable HIV outcomes by attending to diversity: A qualitative study exploring the needs of LGBTQI+ young people living with HIV in Zimbabwe

**DOI:** 10.1371/journal.pgph.0002442

**Published:** 2024-01-25

**Authors:** Joni Lariat, Webster Mavhu, Thandiwe Mudhumo, Pueshpa Shaba, Sharon Sibanda, Rufaro Mbundure, Carol Wogrin, Abigail Mutsinze, Nicola Willis, Sarah Bernays

**Affiliations:** 1 School of Public Health, University of Sydney, Sydney, Australia; 2 Centre for Sexual Health and HIV/AIDS Research (CeSHHAR), Harare, Zimbabwe; 3 Department of International Public Health, Liverpool School of Tropical Medicine, Liverpool, United Kingdom; 4 Zvandiri, Harare, Zimbabwe; 5 Global Health and Development, London School of Hygiene and Tropical Medicine, London, United Kingdom; University of California San Francisco, UNITED STATES

## Abstract

Leaving nobody behind in the fight to end the HIV epidemic as a public health threat depends on addressing inequities in optimal HIV outcomes. Consistently overlooked in research, policy and programming are young lesbian, gay, bisexual, transgender, queer/questioning and intersex (LGBTQI+) people who are living with HIV. This study engaged young LGBTQI+ people in Zimbabwe to better understand their experiences of living with HIV and the support they need. Between September 2022 and February 2023, we conducted qualitative research with 14 LGBTQI+ young people (18–24 years), (two focus group discussions and in-depth interviews with 5/14). All 14 participants were accessing a LGBTQI+ HIV support group at Zvandiri (‘As I Am’), a well-established community-based HIV program. We conducted thematic analysis and key findings informed the collaborative development of internal activities to further enhance inclusivity of LGBTQI+ young people within Zvandiri’s programs. There was consensus among participants that being LGBTQI+ and living with HIV leads to *“double stigma and double trouble”*, involving physical and verbal harassment, social exclusion and family rejection. Participants concealed their LGBTQI+ identity and HIV status in most situations, and many withheld their HIV status in LGBTQI+ social spaces, including community-led LGBTQI+ services. This negatively impacted their psychosocial well-being and social connectedness. Participants described positive experiences of Zvandiri. Interacting with others living with HIV in a destigmatising environment promoted self-acceptance. However, reflecting their prevailing experiences, participants were cautious about revealing their sexuality and/or gender identity at Zvandiri outside of their support group. Ensuring equitable access to HIV care, including mental health support, relies on understanding the challenges experienced by those most marginalised. Critically important is understanding the impact of intersectional stigma on LGBTQI+ young peoples’ social lives, and their access to services. Community-based HIV support programs are well-positioned to support and advance this group’s health rights.

## Introduction

### Pursuing targets: Who is left out?

By 2025, the Global (95-95-95) HIV targets are that 95% of all people living with HIV know their status, 95% of those with HIV are on sustained treatment and 95% of those on treatment are virally suppressed [1]. Although there have been significant advancements towards these targets, progress has been uneven between and within countries, and in some settings, there has been a resurgence in new infections among marginalised groups [2]. This disparity in outcomes has drawn attention to those left behind in current approaches to ending HIV and the persistent, and in some settings, growing inequities in access to HIV care [2]. Subsequently, this has prompted recognition of the need for new strategies to address the social and political barriers that amplify stigma and discrimination, and often criminalisation, that increases marginalised groups’ vulnerability to HIV [3, 4].

Key population groups, which include men who have sex with men, transgender people, sex workers, people who inject drugs and people in prison or detention, receive less dedicated funding and resources to address the structural barriers that create their marginalisation [2, 5–7]. Although HIV prevalence data and other measures are underestimated due to the inaccessibility of services [4], key populations, and especially young people within these groups, are exposed to higher risk across each stage of the HIV care cascade (testing, early treatment, viral suppression) [8–10]. While there has been considerable research worldwide, reflecting the rich regional variability of issues that contribute to poor HIV-related health outcomes for adult key populations [11–14], there are comparatively fewer dedicated qualitative studies focusing on the different challenges and experiences of young people.

There has been a growing emphasis in international guidelines on the different needs of young key populations, including the promotion of greater inclusion within existing youth services [5]. However, there is a lack of general understanding about how young key populations experience and perceive these services. In particular, there is currently little insight into how young key populations’ perceptions and subsequent engagement is shaped by local social and political conditions. Under-recognised too, is the heterogeneity among and within key populations, highlighting the need for greater investment in hearing directly from young people living in a range of contexts.

Lesbian, gay, bisexual, transgender, queer/questioning, intersex and other sexuality, and gender diverse (LGBTQI+) young people’s experiences of stigma and discrimination when accessing health services have been documented in many settings [15–18], with an emerging literature from Southern Africa [19]. Yet, there is very little research from the Southern African region that foregrounds the voices and perspectives of young people, particularly those who are affected by multiple systems of marginalisation, such as those living with HIV and are LGBTQI+. This research responds to this evidence gap.

Importantly, not all sexuality and gender diverse people are classified as ‘key populations’. Lesbian, bisexual, queer and non-binary people, for example, are not included in key population policies and programming, despite their heightened risk of sexual violence in many settings, which significantly increases their risk of HIV and poor HIV-related health outcomes [20, 21]. Due to their invisibility within narrow definitions of risk, these groups are persistently left out of research and subsequent programming. This research responds to these omissions by adopting a more expansive and person-centred approach to ensure equitable representation of the diverse young people who are accessing HIV support services.

### The invisibility of LGBTQI+ young people living with HIV in Zimbabwe

While Zimbabwe has surpassed the 95-95-95 targets in 2022 [22], recent studies have illustrated persistent disparities in HIV outcomes for the country’s marginalised populations [23]. An acutely neglected group are LGBTQI+ young people living with HIV.

Government health services in Zimbabwe, as is common globally, are based upon hetero-cis-normative assumptions, and beliefs and attitudes towards homosexuality and gender diversity present significant barriers to young LGBTQI+ people accessing basic healthcare and HIV management services [19]. Those living with HIV experience intersectional stigma, which arises from the convergence of multiple stigmatised identities, conditions, or behaviours and their interaction with structural factors (such as social norms and legislative and policy frameworks) to impact health [24]. This intersectional stigma exacerbates barriers to access, threatens to disrupt engagement in treatment, and places additional pressures on mental health and well-being [24–28].

Young people are currently ill-served by adult LGBTQI+ dedicated services, which ration their accessibility to young people due to a well-founded fear of negative community reactions [19]. While there has been significant attention placed on improving tailored services for children, adolescents, and young people living with HIV (CAYPLHIV), there has been no attempt yet to understand the differentiated care needs of LGBTQI+ young people within this group. While this diverse group undoubtedly do access community-based HIV peer-delivered support services, attitudes surrounding adolescent sexuality, homosexuality and gender norms intersect to create a silencing that renders them invisible in practice, programming and research.

There are important exceptions to this trend. A leading example of this, and a case study for this research, is Zvandiri (‘As I Am’), a community-based HIV program operating at a national scale in Zimbabwe and in eleven other countries. Their services are delivered by trained and supported community adolescent treatment supporters (CATS), aged 18–24 years who are themselves living with HIV [29–31]. Zvandiri, in Zimbabwe, have recently begun developing interventions to better meet the needs of LGBTQI+ young people accessing their program, including the establishment of a peer-led LGBTQI+ support group. The formation of this group offered an opportune time to engage young LGBTQI+ people who are living with HIV in research to foreground their voices and ideas [32, 33], whilst assuring their safety and privacy. This study aimed to develop an evidence base with young people that could be used to inform internal activities to further enhance inclusivity within Zvandiri’s programs and to support a more nuanced understanding of potential differentiated needs of young people living with HIV that may have conceptual transferability across contexts.

## Methods

### Study design and participants

This qualitative enquiry was ancillary to a larger mixed-methods study, *Zvandiri Character Strength and Its Constructs among Adolescents Living with HIV in Zimbabwe (ZCS)*. Research has recently demonstrated the positive impact the Zvandiri program has on CAYPLHIV’s health outcomes, including viral suppression [34–36]. The ZCS project is primarily concerned with *how* and *why* Zvandiri has this positive effect. This smaller study explored the experiences of LGBTQI+ young people living with HIV who are receiving Zvandiri support.

Participants were recruited from a peer-led LGBTQI+ support group within Zvandiri. To be included in the study, participants had to be aware of their HIV status and be receiving some support from Zvandiri. The support group has a diverse membership of lesbian, gay, bisexual, transgender, and queer young people. Our inclusion criteria aligned with the group’s, enabling us to use convenience-based sampling to recruit participants within a purposively selected group. Reflecting the group’s composition at the time, we were unable to recruit intersex participants. Accessing participants from the support group mitigated the risk of unintentional disclosure [37, 38], as all participants were aware of one another’s sexuality, gender identity, and HIV-status, and knew and trusted one another. Participants all lived in or around Harare, and presented a range of experiences related to HIV disclosure, acquisition, and how long they had been accessing Zvandiri services.

The study design was informed by community-based participatory action research principles [39]. Participants were integral to all aspects of the research, including conceiving the research questions, co-designing data collection methods and research outputs, and contributing to authorship. Researchers (JL, WM, SB) introduced the research concept to the support group’s peer mentor (26-years-old, LGBTQI+ community member and activist) who then worked with the group to distil their priorities and preferences for if and how they would participate in the research. The peer mentor was an important conduit between the group, Zvandiri and the research team. This three-way conversation ensured the group’s interests were driving the research and enabled a responsive and agile study design to mediate concerns as and when they surfaced.

All 12 original support group members enthusiastically agreed to participate. Nine joined focus group discussion-1 (FGD-1). Three were unavailable at the time most suitable to the majority and so participated in FGD-2. The support group had gained two new members by FGD-2, both of whom were recruited into the study to participate in FGD-2. Three attended FDG-1 and FDG-2. We then selectively invited five FGD participants, who represented the breadth of diversity across the group, to participate in in-depth individual interviews (IDIs).

### Data collection

Two FGDs (n = 14 young people 18–24 years-old) were held at Zvandiri between September and October 2022. Over half (57%) were >23 years-old. Five transgender women (36%), four bisexual women (29%), three gay men (21%), one lesbian (7%) and one queer person (7%) participated. FGD-1 included nine participants, FDG-2, eight. Three participants (all aged 24) attended both FGDs. The FGDs ran for 1.5 hours and were co-facilitated by the peer mentor and PS, a trained and experienced local qualitative researcher, familiar with Zvandiri.

Follow-up IDIs were conducted between November 2022 and February 2023 with five of the participants who had attended at least one FGD to allow a depth of exploration on key themes covered in the FGDs, and to cross-check whether what was shared in the FGDs had been curtailed by group dynamics. IDIs were conducted by the same local researcher who conducted the FGDs to ensure continuity and rapport. FGDs and interviews were conducted in both Shona (local language) and English, which was guided by participants, and were recorded, transcribed, and translated by the researcher who conducted the interviews. Transcripts were deidentified and stored in a password protected file on a computer only accessible to the research team.

Flexible topic guides (S1 Text) were used and adapted to fit the method. They covered: stigma and discrimination experiences; impact of stigma on HIV status disclosure, family relations, access to HIV treatment and support services, and mental health and wellbeing; experiences and perceptions of Zvandiri, including how the program has supported them, and what they think could be improved; and their perspectives about their own strengths, resilience, and adaptive capacities.

### Data analysis

Inductive interpretive thematic analysis was conducted to generate themes within the data [40–42]. Three researchers (JL, SB, PS) familiarised themselves with the data as it was collected and produced analytical memos alongside initial coding to iteratively inform subsequent data collection [43]. Codes were collated into themes to capture the deeper sociological issues shaping intersectional experiences among individuals within the group [44]. The research team met weekly online to discuss similarities and differences in coding and thematic conceptualisation. JL and the peer-mentor met throughout the research process to generate deeper insight, contribute to iterative data collection, and to develop outputs, including this publication.

### Ethics

Ethics approval was provided by the Zimbabwe national ethics committee, the Medical Research Council of Zimbabwe (#A/2860). Participants provided written informed consent to participate in the FGDs and IDIs and to being audio recorded, which was witnessed by a research team member and Zvandiri’s Program Coordinator (AM). Participants’ quotations are labelled using their chosen pseudonym, or a participant identification (PID) number if they did not select a pseudonym. Respondents received US$10 bus fare reimbursement.

### Inclusivity in global research

Additional information regarding the ethical, cultural and scientific considerations specific to inclusivity in global research is included in S1 Checklist.

## Results

### “I am a queer person who is just themselves. I have no limits or boundaries”

When invited to share their identities and pronouns, participants described their identity as personal and embodied, fluid and evolving. For some, labels enabled an empowering expression of identity and experience. For others, the available language, and the local contextual ways in which labels are used to denigrate LGBTQI+ people, led them to resist tethering their identity to any one term or category.

### The limits of acceptance

#### “It’s now double stigma and double trouble”

Participants described occupying two stigmatised identities as living with “double stigma” that is shaped by context. Their stories illustrated how acceptance from family, friends and the community is conditional and precarious, and largely depends on their ability to manage what others know about them. This led many to hide their HIV status and LGBTQI+ identity to protect themselves and their relationships.

‘You have to live a double life. I can be myself in my house alone, but outside, no matter how much I need to go out to get help, I can’t open up because I don’t know the reaction that I am going to receive’

(Strawberry, IDI 1).

“Double stigma” was described as accumulating throughout adolescence and young adulthood. The usual stressors associated with adolescence felt amplified and compounded when participants became aware of their sexuality and/or gender identity and HIV status, largely because of others’ anticipated reactions. This following quote illustrates this internal instability:

‘You are a young person, right? And you have issues about your sexuality that you are already dealing with. And you haven’t found comfort and it’s troubling. You are also being talked about. So now you have this new issue as well. You are already scared and it’s now double stigma and it’s double trouble. You don’t know where to take it’

(PID 05, FGD-2).

Fear of others’ responses led many participants to withdraw from social situations or conceal parts of their identity. This rationing of contact with others, even in accessing support, further exacerbated their social isolation, confusion, self-stigma, and loneliness.

‘I didn’t just wake up like, “Well, I am who I am, and I like it”. It stressed me out because I felt there is something wrong with me and I don’t understand what the hell is going on with me right now. There comes a time when you need to express yourself to others. But then rejection comes. I don’t go [to places for support] because I have this fear that people will see me, and I don’t know how they are going to take it’

(PID 01, FGD-1).

Family rejection and the consequent risk of homelessness was most concerning for participants. Family members’ negative reaction to participants’ HIV status were often inflected with homophobia grounded in religious beliefs that homosexuality is satanic and punishable by HIV infection.

‘Some of us, we were born with it (HIV) but now people start judging us saying, “Do you think God loves it? They are being punished by God because of being LGBTQI+. God punishes people. There’s no way He will fail to punish these”‘

(MG, FGD-1).

Anticipated homophobia and blame led several participants to keep their identity and status a secret from family.

‘For my parents to understand that I got HIV from another man is challenging. So, it’s better to be quiet’

(PID 01, FGD-2).

One participant described how, for her, the assumption that being LGBTQI+ makes HIV infection inevitable flowed in an inverse direction. She acquired HIV perinatally and when she came out as lesbian during early adulthood,

‘People said, “That child has changed because of HIV. She is now reckless. Have you noticed she is now a lesbian because she was born HIV positive?” So, the community concludes that just because you are HIV positive, you have chosen to be lesbian’

(PID 08, FGD-2).

As this quote illustrates, compounded negative responses falsely associate a causative relationship between HIV status and homosexuality. Whichever (sexuality or HIV status) was known first was attributed as causing the other and subsequently, for explaining the nature of the individual’s ‘spoilt’ identity.

Several participants felt responsible for protecting their family’s reputation by keeping their sexuality secret. This was intensified for those whose family had experienced stigma for having a child born with HIV. Others experienced the inverse, where their gender non-conformity was recognised by the community, and family members sought to avoid further stigmatisation by pressuring their child to conceal their HIV status, as in this example:

‘When I was diagnosed with HIV, I told my mom. She said, “you don’t have to tell the family”. Already the family discriminated against me because I was born different’

(PID 07 FGD-2).

#### Concealment is not an option for everyone

Experiences of stigma and discrimination were influenced by gender expression and how visible their identity is to others.

‘Certain groups are discriminated more because they are doing something loud. It’s something that you can’t avoid because of the way you talk, dress and even walk’

(PID 06, FGD-2).

For the trans women participants, gender-affirming clothing was critically important to their sense of self and well-being; however, it increased the likelihood of harassment and violence.

‘I am a transgender person. I like to go out in nails, earrings and artificial hair and I will be feeling comfortable in that, with what I am wearing. The moment I step into the public clinic, all eyes are on me because I am different… I might end up being beaten up’

(Izinduna, FGD-1).

Those who proudly expressed their gender in healthcare settings described receiving open judgement, hostility and invasive questioning from healthcare workers. A lack of confidentiality and privacy were also common.

‘They [healthcare workers] say, “Look, what a disgrace” because when you go there, some of us we can’t pretend. When I go to collect my ARV supply, they ask, “How did you acquire HIV?” They will be saying, “Obviously you don’t have a girlfriend because of your looks”. They have already judged you: This person is gay. And they start calling each other to come and see you’

(PID 02, FGD-1).

These incidents strongly dissuaded participants from attending public clinics. Some had learned, through personal experience and information-sharing, which clinics were safest, and where they might find ‘sensitised’ staff.

Despite these challenges, participants rarely went without their medication resupply, sourcing it from LGBTQI+ organisations, private clinics, or having someone pick it up for them. One participant utilised Zvandiri as a safe space to take their daily medication. When participants did attend public clinics, they often ensured a known CATS (Zvandiri peer-supporter) would be present, which significantly eased interactions with staff.

‘I don’t want the people to ask me things, so I call the CATS to say that I am coming. He will look for my book and be waiting for me. From the gate to the office, we walk and get medication while talking and I will be smiling, no stress. No one will even ask me anything. He will be doing the process’

(PID 05, FGD-2).

#### “Our community leaks”

Participants described having few opportunities to openly express their sexuality and/or gender identities. LGBTQI+ organisations offered a chance to meet other LGBTQI+ young people and are important places to access non-judgmental sexual and reproductive health care. However, participants also described feeling that they must keep their HIV status hidden in these spaces to avoid reputational damage and rejection.

‘In these places for the LGBTQI+ community, I am only free to express myself as a LGBTQI+ person. There is nowhere safe in this world that you will stand to say, “I am HIV positive”‘

(PID 01, FGD-2).

There are dangers in revealing their HIV status to peers and intimate partners, but also peer-educators who work for LGBTQI+ organisations. Knowledge of someone’s HIV status appeared to be traded like a social currency within LGBTQI+ social networks, with personal information quickly shared through instant messaging groups and social media.

‘In our community the gossip spreads so fast, just like fire lit on the grass. Just by telling your peer, the information will spread. By evening everyone would have been informed and now, because of social media, the message will spread in the groups and that will affect my mental health’

(PID 07, FGD-2).

The contradiction of being accepted within the LGBTQI+ community, but actively excluded when their HIV status became known, was evidently destabilising for participants. The following passage is illustrative of the tension between the desire to seek support and acceptance whilst remaining alert to the dangers of disclosure.

‘I might go there [LGBTQI+ organisation] for testing and collecting my medication, but then I might be invited to a social event. You feel encouraged when you are there that it’s a safe space, but we forget that there is reality after that place. You get carried away because you have a zeal to open up, you have this thing that is burning, and you want to let it out. You think, let me just try and say something so that I can relax my head and my heart. When you get home, your phone will be bursting with calls and texts by people saying, “We heard that you said so and so, why didn’t you tell us all along that you are like that?”‘

(PID 05, FGD-2).

### Social spaces where I can be (seen as) all of me

The discrimination participants described was contrasted with rare instances of support and acceptance. For one participant, an aunt’s acceptance provided a welcoming and safe space for them and their friends to spend time. For another, rejected by their family, a friend’s home offered a temporary space to explore independence and a growing sense of self.

These, however, were uncommon experiences. Most participants described not having anywhere they felt completely free to express their identity and be open about their HIV status. Even at Zvandiri, outside of their immediate LGBTQI+ support group, some participants were wary of the risks of being openly LGBTQI+: ‘if you ask me is there a place where I feel safe? I don’t have’ (PID 02, FGD-2).

Yet, feeling confident to be open about their HIV status at Zvandiri, a rare space where HIV is normalised, enabled them to access more optimistic ways of thinking about their HIV status. For some, this had flow-on effects for confidence and acceptance of their LGBTQI+ identities.

The benefits of attending Zvandiri largely centred on the affirmation, reassurance and motivational effects of interacting with other young people living with HIV.

‘Before coming to Zvandiri, you wouldn’t know that there are young people living with HIV just like you. When you start attending support groups, you start to feel at home. It’s different from the outside where there is stigma and discrimination’

(MG FGD-1).

Some participants emphasised that Zvandiri’s support extended beyond HIV into other aspects of their lives. Several older participants had developed strong relationships with individual CATS, where they received psychosocial support in addition to practical assistance. They described experiencing a growing sense of optimism and social safety through their participation in support groups, which for some, ignited their own feeling of being a support for other beneficiaries.

‘For me, it’s the support groups that I enjoy more than anything else. We come and share our mindsets and our life experiences, and we get to strengthen and edify each other’s lives. You know, we wouldn’t only talk about HIV and how to live, but we would talk about so many things’

(Strawberry, IDI 1).

That everyone accessing the program is living with HIV imparts a relational accountability in social interactions that your status will not be disclosed beyond Zvandiri.

‘I like that people in Zvandiri are living with HIV. It makes me feel comfortable because I know that person won’t leak the information’

(PID 04, FGD-2).

Witnessing others face similar challenges has an immensely positive effect on self-acceptance.

However, the assumed heterosexuality of beneficiaries, the binary logic underpinning support groups, and Zvandiri staff’s lack of knowledge in how to respond to their different needs, negatively impacted some participant’s engagement. As one participant explained:

‘I think I attended two support groups. I was the only one [LGBTQI+ person], and they have little or no knowledge of how to deal with this… they will just be discussing things like how to approach your boyfriend or girlfriends, so I felt left out and I saw that these issues had nothing to do with me and decided to stop attending’

(PID 2, IDI 05).

#### The coming together of an LGBTQI+ support group—Fleeting moments of Zvandiri

The Zvandiri LGBTQI+ support group emerged from the recognition of a growing need for a safe space for LGBTQI+ beneficiaries to meet and access peer-support. For several participants, this reignited their relationship with Zvandiri and initiated a transformation of their self-esteem and self-acceptance.

‘In this support group we find time to see each other and talk. It builds my confidence. It makes me accept who I am’

(KM, FGD-1).

LGBTQI+ group meetings at Zvandiri were described as a rare opportunity to openly express all aspects of their identity. Witnessing others allowed mutual problems and understanding to be shared and the intersectionality of living with HIV as a young LGBTQI+ person to emerge.

‘I might have this huge story and someone else is also going through it too. You get to understand yourself through other people. Now I am coming to a space where I am finding HIV positive people, LGBTQI+ community, people that experience similar problems as mine and they are surviving, thriving, and working on being better people despite HIV and all’

(PID 04, FGD-1).

#### “Zvandiri is talking to me”

Although many participants did not feel confident in revealing their LGBTQI+ identity beyond their own support group, they identified with Zvandiri’s foundational principles of self-acceptance of HIV status (Zvandiri means ‘As I am’ in Shona).

‘When I heard the name “Zvandiri” I knew it was talking about me. It’s called Zvandiri (as I am) not *zvavari* (as they are). I knew this is where I belong because it’s talking about me, whether I am gay or lesbian or HIV positive. “Zvandiri”: this is me’

(PID 07, FGD-2).

This perspective was widely shared across the sample, indicating that Zvandiri represents more than a physical location to visit, or a static concept to fit into. It suggests that participants feel included and ‘spoken to’ by the service, even if there are still spaces within Zvandiri where they are not yet comfortable to openly express their sexuality and/or gender identity. Unlike anywhere else, Zvandiri offers the strongest possibility that they do not have to pretend to be someone else (as *they* are) to be accepted.

Participants shared suggestions for how Zvandiri could make this inclusion explicit to help realise their fuller participation. They emphasised the need for staff and CATS to develop greater awareness of LGBTQI+ young people, their needs and challenges, through ‘sensitisation’ training, developed by LGBTQI+ young people.

‘Sensitisation should happen while we are there, not when we are not around, with others saying, “LGBTQI+ people are like this, go and treat them like that”‘

(PID 05, FGD-2).

‘We should be there, presenting while wearing the actual clothes I like to wear, and they will know we truly exist’

(PID 06, FGD-2).

## Discussion

### Attending to diversity and responding to intersectional stigma

This study highlights the need for greater recognition of the sexuality and gender diversity that exists among CAYPLHIV who are accessing community-based HIV support programs in regions of Africa with high HIV prevalence among young people. Inequities in optimal HIV outcomes are perpetuated by the failure to recognise the rich diversity of CAYPLHIV across a range of intersecting identities and experiences.

As participants’ accounts in this study demonstrate, the need to conceal parts of their identity to protect themselves against stigma and discrimination has significant detrimental impacts on mental health and well-being. It also renders LGBTQI+ young people invisible in most settings, including in spaces where HIV-stigma is alleviated, such as in community-based HIV support programs like Zvandiri, which perpetuates the myth of adolescent homogeneity. Improving access and engagement in HIV care depends upon enhancing these spaces to ensure all young people are able to safely express their full identities.

This study also reinforces recent scholarship emphasising the importance of understanding the impacts of intersectional stigma on adolescent’s engagement with HIV services [45] and current efforts to better integrate intersectionality in stigma reduction interventions [46].

Stigma is pervasive and affects many people living with HIV [47, 48]. While the accounts of stigma described in this study might feel familiar for those well-versed in the adolescent HIV literature, our analysis highlights that HIV-stigma often manifests differently, and with varied implications for social participation, for LGBTQI+ young people. This is because the public perception of their non-normative sexuality and/or gender expression mediates societal responses to their HIV status. Participant’s accounts clearly illustrate that the HIV-stigma they experience is compounded by the discrimination they face for being LGBTQI+: homophobia and transphobia inflects, and is inflected by, HIV-stigma to create specific experiences of discrimination and marginalisation for those who exist within this intersection. Better understanding this complex interplay of stigma is crucial for designing effective and appropriate psychosocial support strategies for this group. These nuanced insights are also vital if efforts to challenge systemic and structural stigma and discrimination are to be effective in reducing barriers in access to HIV testing, treatment and care for marginalised young people.

Consistent with community-based participatory action research principles, these early insights are informing immediate and long-term strategies to enhance inclusivity at Zvandiri as a first step in improving the mental health and wellbeing of LGBTQI+ young people accessing the program. For example, following a research sharing event designed by participants in which they delivered a creative representation to selected Zvandiri staff and managers, efforts to increase LGBTQI+ representation among CATS (Zvandiri peer-supporters) has begun. This was critical, as part of Zvandiri’s endeavour is that their peer to peer model is responsive to the complex intersectionality of identities within Zvandiri through the delivery of differentiated services [36, 49, 50]. We have also begun co-designing training resources with participants and Zvandiri to equip staff and peer-supporters with the knowledge and skills to better support LGBTQI+ young people. This is being shaped by the young people, with spaces created for open dialogue through which Zvandiri can learn and collaboratively develop advocacy, training and differentiated service delivery for LGBTQI+ at scale with young people. Indicative of the youth-led approaches being adopted, is the emerging focus on the celebration of creativity with positive psychology (focuses on the positive events and influences in life) being central to the development of tailored psychosocial support.

### The need for a strengths-based intersectional approach to delivering differentiated care within community-based peer-led HIV support programs

Participant’s accounts in this study demonstrate the diverse experiences, challenges and strengths within the young LGBTQI+ community in Zimbabwe. This is reflective of the broader diversity among adolescents and young people who are living with HIV. We must resist homogenising LGBTQI+ young people, as they often are when we use terms like ‘key populations’ to guide HIV prevention and treatment service delivery. Greater investment in person-centred care is needed to counter these tendencies and to extend appropriate support to all CAYPLHIV. Youth-centred programs such as Zvandiri, that already centre principles of inclusivity and acceptance, are most amenable to incorporating intersectionality in the design and implementation of their programs.

An intersectional framework also supports greater recognition of the adaptive capacities and social support networks that stigmatised communities develop to protect and support themselves [28]. It is critical that we recognise how LGBTQI+ young people living with HIV already support themselves and one another so that we can adapt and develop care models to be effective within existing care networks. In this study, we witnessed this group’s innovation to come together to support one another. These social networks and ways of making family and community necessarily differ from what we know about the community and family support systems of non- LGBTQI+ CAYPLHIV, many of whom also have diverse care arrangements [51, 52]. Further research into the similarities and differences will facilitate appropriate engagement of young LGBTQI+ peoples’ support networks in wrap-around care [53].

There is also great potential in strengthening the advocacy role already played by community-based HIV support programs for CAYPLHIV. For LGBTQI+ beneficiaries, a step towards improving their lives would be redressing homophobia and transphobia within their families and communities. As the group emphasised, their greater inclusion in society would have a significant positive effect on their health, well-being and capacity to engage in social and economic activities. This is an endeavour community-based support programs like Zvandiri are strategically positioned to lead because they are trusted by the community and are already playing an important role in contesting HIV misinformation and stigma. There is scope then to intentionally engage with respected community and religious leaders to demonstrate the importance of greater inclusion of LGBTQI+ young people in general and to those living with HIV in particular.

As emphasised within young people’s accounts in this study, we need to evaluate the language we use to describe marginalised groups and communities. Their accounts of intersectional stigma emphasise that their needs cannot be reduced to behaviour, as is implied in the categorisation of key populations according to perceived risk. Additionally, not all LGBTQI+ people are classified as key populations, leading to the persistent invisibility of some highly marginalised and structurally vulnerable groups. Using language that targets discrete groups, such as ‘MSM’, is inadequate to encapsulate the rich diversity of Southern Africa’s young flourishing LGBTQI+ community. Tailoring affirming and inclusive programs to LGBTQI+ young peoples’ needs, not their behaviours, within existing community-based peer-led HIV support programs, will reaffirm LGBTQI+ young peoples’ sense of belonging in these spaces.

## Limitations

Given the scarcity of research centring LGBTQI+ young people who are living with HIV in high HIV-prevalence African countries, the data we were able to collect with a small group of young people generated insights for pursuing improvements in the health and well-being of this marginalised and under-served group. There is great scope and need for gaining deeper insight into the diversity of LGBTQI+ young people living with HIV in Southern Africa, and to ensure greater representation of the issues facing intersex young people.

## Conclusions

LGBTQI+ young people who are living with HIV in Zimbabwe currently fall through gaps in policy, healthcare interventions and research. Community-based peer-led HIV support services, like Zvandiri, are an amenable setting for improving inclusivity of LGBTQI+ young people. Efforts to explicitly include the needs of this group in programming, and providing training for staff and peer support volunteers, informed by LGBTQI+ young people themselves, should be prioritised. Where training will likely be most transformative is in deepening awareness and understanding of how stigma impacts young people who exist within the intersections of multiple stigmatised identities.

‘Leaving nobody behind’ requires addressing inequities in access to HIV care, including differentiated mental health care and support. We must take seriously our responsibility to pay closer attention to diversity among young people, to pursue deeper understandings of those overlooked in the HIV response, and to advocate for their greater inclusion.

## Supporting information

S1 Text Facilitator focus group discussion and individual in-depth interview flexible topic guides. (PDF)

S1 Checklist Inclusivity in global research questionnaire. (DOCX)
