# Supplementary material for: Leaving no one behind? Addressing inequitable HIV outcomes by attending to diversity: A qualitative study exploring the needs of LGBTQI+ young people living with HIV in Zimbabwe
Source: PLOS Glob Public Health. 2024 Jan 25;4(1):e0002442. doi: 10.1371/journal.pgph.0002442 (PMC10810535; doi:10.1371/journal.pgph.0002442)
Supplement: S1 Text — (PDF) [file pgph.0002442.s001.pdf]

LGBTQI+ Zvandiri Support Group:
A sub-study within *Zvandiri Character Strength and Its Constructs among Adolescents Living with HIV in Zimbabwe*.

Facilitator Focus Group Discussion and Individual In-depth Interview Flexible Topic Guides

Focus Group Discussion 1:

Topic	Framing/prompts/questions
**Introducing the project, research team, and the purpose of today's discussion**	• Research team co-facilitator to introduce the research objectives and respond to questions.<br>• Research team to introduce and explain informed consent process.<br>• Opportunity to choose a pseudonym.<br>• Sign consent forms.
**Introductions**	• For those who feel comfortable, would you like to start by introducing yourself?<br>- You could share how old you are, how long you have been coming to Zvandiri, how you describe your sexuality and/or gender identity.<br>- You can include any details you would like us to know about your identity.
**Terminology**	• The words people use to describe their sexuality and gender identity changes from place to place. Young people might describe themselves differently to older people in the same community.<br>• Are there local terms that young people prefer to use that you would like the researchers to use?
**Experience of Zvandiri**	• How long have you been coming to Zvandiri?<br>• Can you describe how supported you felt before and then after coming to Zvandiri?<br>• Has Zvandiri helped you with challenges you face?<br>• How could Zvandiri be improved to support your specific challenges?
**Experience of support group**	• What does this support group mean to you?<br>• How have you felt since joining the group?<br>• Has the support group helped you with challenges you face in your day-to-day life?
**Experience of healthcare services**	• What challenges have you faced when accessing healthcare?<br>• What would you like to change about how healthcare is currently delivered to you?<br>• What do you think the positive impact of these changes would be?
**Conclusions/ wrapping up**	• Is there anything we haven't spoken about that you would like to add?

Focus Group Discussion 2:

Topic	Introduction of topic	Example questions
**Stigma towards people living with HIV in the LGBTQI+ community – impact on disclosure and access to services.**	In the first FGD, several people mentioned that people living with HIV face stigma and discrimination in the LGBTQI+ community.<br><br>Could we discuss that more?	• *What are your main concerns about what could happen if people in the LGBTQI+ community know about your HIV status?*<br>• *How does this impact whether you feel able to disclose your status in your relationships?*<br>• *Do you feel that you can disclose your status to providers in LGBTQI+ organisations or to other young people who access these services?*<br>• *Last time, some of you described the lack of privacy and confidentiality at public clinics.*<br>• *Are there similar issues with privacy and confidentiality about HIV status in LGBTQI+ organisations?*
**Stigma in other settings – home and church**	Last time we discussed the kinds of misinformation that exists about HIV and LGBTQI+ identities in the community. Some of you said that you cannot be yourself at home and church – can we talk a bit more about that?	• *Am I right in understanding that you cannot be open about your HIV status or being LGBTQI+ with people at home and at church?*<br>• *What could happen if people find out about your sexual orientation?*<br>• *Some of you mentioned last time that you have nobody at home that you can speak to about your issues – especially about your sexual orientation.*<br>• *How did this impact your ability to manage your HIV treatment – for example, did it impact you taking your medicine or going to your reviews?*<br>• *How do you think it impacted other things, like going to school or your friendships?*<br>• *Do you get emotional support from family? Your church community? Or is it difficult to be open about your challenges with HIV at home/church?*<br>• *Are there any other places, apart from the support group, where you feel you can be open about your HIV status and being LGBTQI+?*<br>• *What would it mean if you could be your full self at home?*<br>• *How would having family support help?*
**What role has Zvandiri played, if any, in supporting you through difficult times?**	In our last meeting, several of you told us about difficult times when you have struggled with your identity or faced negative attitudes from people in your communities.	• *When you have been having a really difficult time, what has helped you?*<br>• *How has Zvandiri played a role in that?*
**Experiences of healthcare services / healthcare workers**	In the last group discussion, some people said that they had missed their resupply or not received STI testing because of the stigma they	• *How often would you say you have missed your resupply or not received STI testing because of stigma at the clinic?*<br>• *If you have been unable to pick up your resupply, have you then felt able to share this with CATS or other Zvandiri staff?*

	experienced at the clinic from healthcare staff.	• *If you were able to tell them, was Zvandiri able to help? How?*<br>• *Regarding STI testing – if you were unable to get an STI test at the public clinic, were you able to receive testing somewhere else?*<br>• *How many people currently feel that they have a healthcare worker at their clinic who they feel safe and comfortable with and who knows about your sexual orientation or your being transgender?*<br>• *How did you find out about them?*<br>• *How long did it take for you to tell them you are LGBTQI+?*<br>• *Do young people share information (for example, on WhatsApp) – how do you know which clinics/ individual HCWs are safe?*<br>• *Are LGBTQI+ orgs (like GALZ) helpful in giving you advice about where to go? How could they be more helpful?*
**Zvandiri, CATS, & the support group**	In the last FGD, several people spoke about how important Zvandiri had been for not feeling so alone. Many also shared that this support group has made you feel supported and confident about being LGBTQI+ and living with HIV.	• *Could we revisit this topic and hear perhaps from people who didn't yet have a chance to share?*<br>• *How has Zvandiri and this support group helped you at home, at church, in your relationships and in the community?*<br>• *Can anyone give an example of a good experience you had at the clinic because a CATS volunteer was there? How did they help?*<br>• *Has there been a time where you would have left without your medication or got an STI test, but the CATS' assistance meant that you stayed?*<br>• *Have you felt able to disclose your sexual orientation or gender identity to a CATS you have interacted with?*
**'Sensitizing' all healthcare staff, from the gateman to the sister in charge**	Last time the group was very passionate about the need to sensitize healthcare staff and the broader community about LGBTQI+ issues.<br>Can we discuss this a bit more?	• *What do you think should be involved in this sensitization process?*<br>• *What would you want HCWs and Zvandiri staff to know/understand about LGBTQI+ young people living with HIV?*<br>• *We know that Zvandiri means "accept me as I am" – what does that mean for LGBTQI+ young people living with HIV?*
**LGBTQI+ strengths – what can others learn from you?**	In the last FGD, we heard so many stories from you that showed how strong and resilient you all are.	• *What do you think are your strengths?*<br>• *What do you think Zvandiri can learn from you?*<br>• *What do you think this group can teach other young people who access Zvandiri?*

Individual in-depth interviews:

Topic	Rationale	Introduction and framing of the topic	Example questions	Follow-up questions and prompts to push a little further
**Introducing experiences of Zvandiri**	Beginning on comfortable ground – easy to answer questions that start with Zvandiri.<br><br>Participants are expecting to discuss Zvandiri, so this is a good place to start to set them at ease. This will be, after all, how you have explained the interview to them at the consenting stage.<br><br>Positive framing.<br><br>If this interview is with somebody you have already interacted with in a FGD, make sure to recall some of what they already shared – can you start with referring to something you know about them – for example, I recall you saying you have been coming to Zvandiri for 4 years – can you tell me a bit more about how you started coming?	If we could begin by asking you some questions about Zvandiri – I would like to get an idea about what it is like to be a part of the Zvandiri community – and to know a little about the activities you have enjoyed since joining.	To start us off, could you tell me a little about how long you have been coming to Zvandiri and how you found out about it?<br><br>How did it feel when you first came? Prompt: were you nervous or excited?<br><br>What parts of Zvandiri do you find most fun/interesting? What do you look forward to the most?<br><br>Was it easy to make friends? How did that feel? Was it different in any way to your friendships with people outside of Zvandiri? How? Why do you think that might be?<br><br>And how about the CATS? How do you find talking to them? What do you talk about? Is that unusual for you? Have the chats that you have had with the CATS changed over time? In what way?	How often do the CATS visit you?<br>Has that been the same since you joined?<br><br>How often do you attend support groups or participate in other activities run by Zvandiri?

**Inviting reflection on experiences prior to joining Zvandiri**	In this section, we want to invite participants to reflect on how the positive experiences they share in the first section, might compare to their experiences prior to joining Zvandiri.<br><br>This section is really in conversation with the previous section – so you will rely on what they have said about their initial experiences of Zvandiri and pose many of the questions in response to what they have already told you.	We have talked a bit about what it is like being involved in Zvandiri. Can you tell me what it was like before you were? Did you have any similar conversations with anyone else other than the CATS about what you were going through?<br><br>We have talked about how your CATS talks to you about different aspects of HIV. Did anyone else do that beforehand? What was that like? Is it different for you when the CATS does it? Why?	*As the questions will be in response to what they have told you- these are just examples. They would not necessarily be relevant to ask to everybody. We would expect that there would be many other questions that should or might be asked.*<br><br>It sounds like you have made some good friends at Zvandiri. How do these friendships differ from your friends who don't attend Zvandiri?<br><br>You mentioned that you have felt happy in the support groups because there are other young people like you, is this a new feeling or are there other people/spaces where you also feel happy?	
**Life around Zvandiri**	We want to get a sense of how Zvandiri interacts with participant's particular social and relational lives.	When you are at a support group/ engaging with Zvandiri, everyone's HIV status is known and you told me that this feels like xxxxx. Can you help me understand,	Earlier you shared that the CATS help you to feel confident – do these feelings stay with you when the CATS aren't around?	

	This section will be a good place to seek out examples or scenarios.	what it is like outside of your activities with Zvandiri? Is it a secret? How do you feel about that? Would you like that to be different? Do you think that it could be? What would have to change for that to happen?		
**Effect of Zvandiri**	Here we want to come back to a reflection on the Zvandiri character strengths, through a prism of what they have shared previously.<br><br>This might only get so far in the first interview – we will follow it up again in the next.<br><br>It is also good to end on a positive and affirming note, after sharing difficult stories.	Thinking about how you have described being involved in Zvandiri and what it was like before you were, can you help me understand what effect, if any, do you think that Zvandiri has had on your life? On you?		If you had two photos of you- one before you joined Zvandiri and one now- would you notice any difference in the two people in the photos?<br><br>How about the bits that can't be seen, but what you know is going on in your thoughts and your heart?<br><br>You have talked about how there is a difference in the way that you feel when you're with people from Zvandiri compared to when you're on your own or with people who don't know. Do you think that there are certain things that Zvandiri won't be able to change for you? Can you tell me more about this?

				When you imagine your future self in five years' time, can you tell me what it looks like in your mind? Do you think that it would look any different if you had not ever got involved with Zvandiri?
